# The effect of the total small vessel disease burden on the structural brain network

**DOI:** 10.1038/s41598-018-25917-4

**Published:** 2018-05-10

**Authors:** Xiaopei Xu, Kui Kai Lau, Yuen Kwun Wong, Henry K. F. Mak, Edward S. Hui

**Affiliations:** 1 Department of Diagnostic Radiology, Li Ka Shing Faculty of Medicine, University of Hong Kong, Pokfulam, HKSAR, China; 2 Department of Medicine, Li Ka Shing Faculty of Medicine, University of Hong Kong, Pokfulam, HKSAR, China; 3 State Key Laboratory of Brain and Cognitive Sciences, University of Hong Kong, Pokfulam, HKSAR, China

## Abstract

Different cerebral small vessel disease (SVD) lesion types have been shown to disrupt structural brain network individually. Considering that they often coexist, we investigated the relation between their collective effect using the recently proposed total SVD score and structural brain network on MRI in 95 patients with first transient ischemic attack (TIA) or ischemic stroke. Fifty-nine patients with and 36 without any SVD lesions were included. The total SVD score was recorded. Diffusion tensor imaging was performed to estimate structural brain connections for subsequent brain connectivity analysis. The global efficiency and characteristic path length of the structural brain network are respectively lower and higher due to SVD. Lower nodal efficiency is also found in the insular, precuneus, supplementary motor area, paracentral lobule, putamen and hippocampus. The total SVD score is correlated with global network measures, the local clustering coefficient and nodal efficiency of hippocampus, and the nodal efficiency of paracentral lobule. We have successfully demonstrated that the disruption of global and local structural brain networks are associated with the increase in the overall SVD severity or burden of patients with TIA or first-time stroke.

## Introduction

Cerebral small vessel disease (SVD), one of the most prevalent neurological disorders, affects small arteries, arterioles, capillaries and small veins^1^, and plays a key role in stroke^2^, dementia^3^ and aging^4^. It is the most common cause of vascular dementia^5^ and accounts for approximately 20% of all strokes^2,6^. SVD has also been implicated in cognitive decline after stroke^7^. Parenchymal lesions on MRI that are considered as biomarkers of SVD include white matter hyperintensities (WMHI), cerebral microbleeds (CMBs), lacunes and visible perivascular spaces (PVS)^4,5,8^, each corresponding to different form of SVD^8^. As these neuroimaging biomarkers of SVD often coexist^9–11^, a total SVD score was recently proposed^12^ to account for the collective effect of WMHI, lacunar infarcts, CMBs and perivascular spaces in a hope to better assess the overall severity or burden of SVD. The total SVD score could also be useful for baseline stratification^11^. It has been validated in stroke patients and healthy population^11–13^, and was found to be associated with the risk factors of SVD^11,12^, cognitive functions^13–15^ and the risk of recurrent stroke^16^.

Considering the sporadic nature of SVD lesions, conventional analyses that are limited to the investigation of regional brain changes^17–19^ are not adequate for the systematic assessment of the global and local effects of these lesions. Given that the brain is a network of interconnected regions, a data analysis known as brain connectivity analysis^20^ was developed so that global and local networks could be systematically and quantitatively characterized^21^. Recent studies using this analysis have shown that the presence of WMHI^22,23^, lacunar infarcts^23,24^ and CMBs^25^ were found to correlate with brain network alterations individually. That the relation between the overall SVD severity and the change in the structural brain network for patients with first transient ischemic attack (TIA) or ischemic stroke is lacking motivates this study. We therefore aim to investigate (1) the extent of the disruption of global and local structural brain networks due to SVD; and (2) the association between the former and the overall severity of SVD.

## Results

Only the findings that are significant will be described below due to the large number of statistical tests, unless otherwise stated.

### Demographics

Patient demographics and SVD findings together with the statistical significance of group statistics are shown in Table 1. There is no significant difference in stroke classification, infarct size and location, and baseline vascular risk factors between patients with and without SVD. Stroke classification, infarct size and location are not associated with any of the vascular risk factors and total SVD score. 59.3% of all patients with SVD had high-grade PVS, 57.6% CMBs, 35.6% high-grade WMHI, and 15.3% lacunar infarcts. The number of patients with total SVD score from 1 to 4 are 30, 19, 8 and 2, respectively. There is no association between age and total SVD score.

**Table 1:** Patient demographics.

	Patients without SVD	Patients with SVD				P-value
Sample size	36	59				
Age	64 ± 11	67 ± 10				0.166
Male gender (%)	39	53				0.196
Prevalence (%)						
Diabetes mellitus	22	24				0.866
Hypertension	44	42				0.843
Ischemic heart disease	6	9				0.597
Ischemic stroke	33	32				0.909
Stroke classification (%)						0.817
Large artery antherosclerosis	33	32				
Small vessel disease	9	20				
Cardioembolism	25	16				
Undetermined	33	32				
Infarct location (%)						
Basal ganglia	50	68				0.305
Brain stem	33	11				0.174
Cerebellum	0	11				0.510
Frontal lobe	8	16				1
Parietal lobe	8	11				1
Temporal lobe	8	5				1
Occipital lobe	0	5				1
Infarct size (cm3)	5.4 ± 12.3	4.5 ± 8.8				0.822
Smoking	14	24				0.245
Antiplatelets	8	17				0.236
Lipid-lowering drugs	17	22				0.526
Serum total cholesterol (mmol/L)	4.7 ± 1.5	4.2 ± 1.6				0.166
		Total SVD Score				
		1	2	3	4	
Sample size		30	19	8	2	
Prevalence (%)						
Lacunar infarct	—	3	16	38	100	
CMB	—	53	47	88	100	
High grade WMHI	—	17	47	75	100	
High grade PVS	—	27	90	100	100	

### Global and local structural brain networks

**Figure 1:**
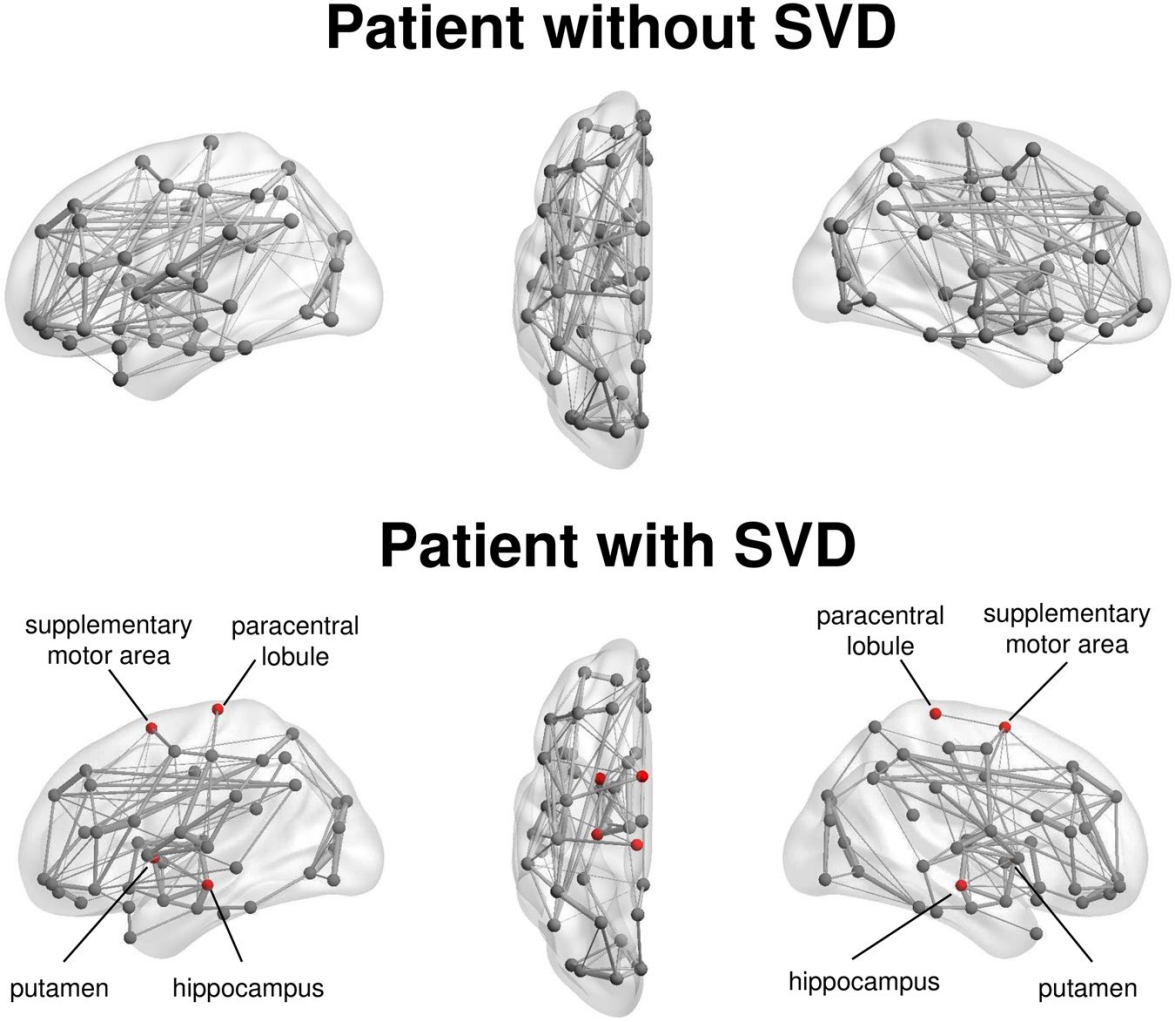
Illustration of the group-averaged structural brain network of patient with first transient ischemic attack (TIA) or ischemic stroke without (top row) or with (bottom row) small vessel disease (SVD). The brain regions with significantly lower nodal efficiency due to the presence of SVD were indicated as red. The node size and edge width are respectively weighted by nodal efficiency and number of connections.

**Figure 2:**
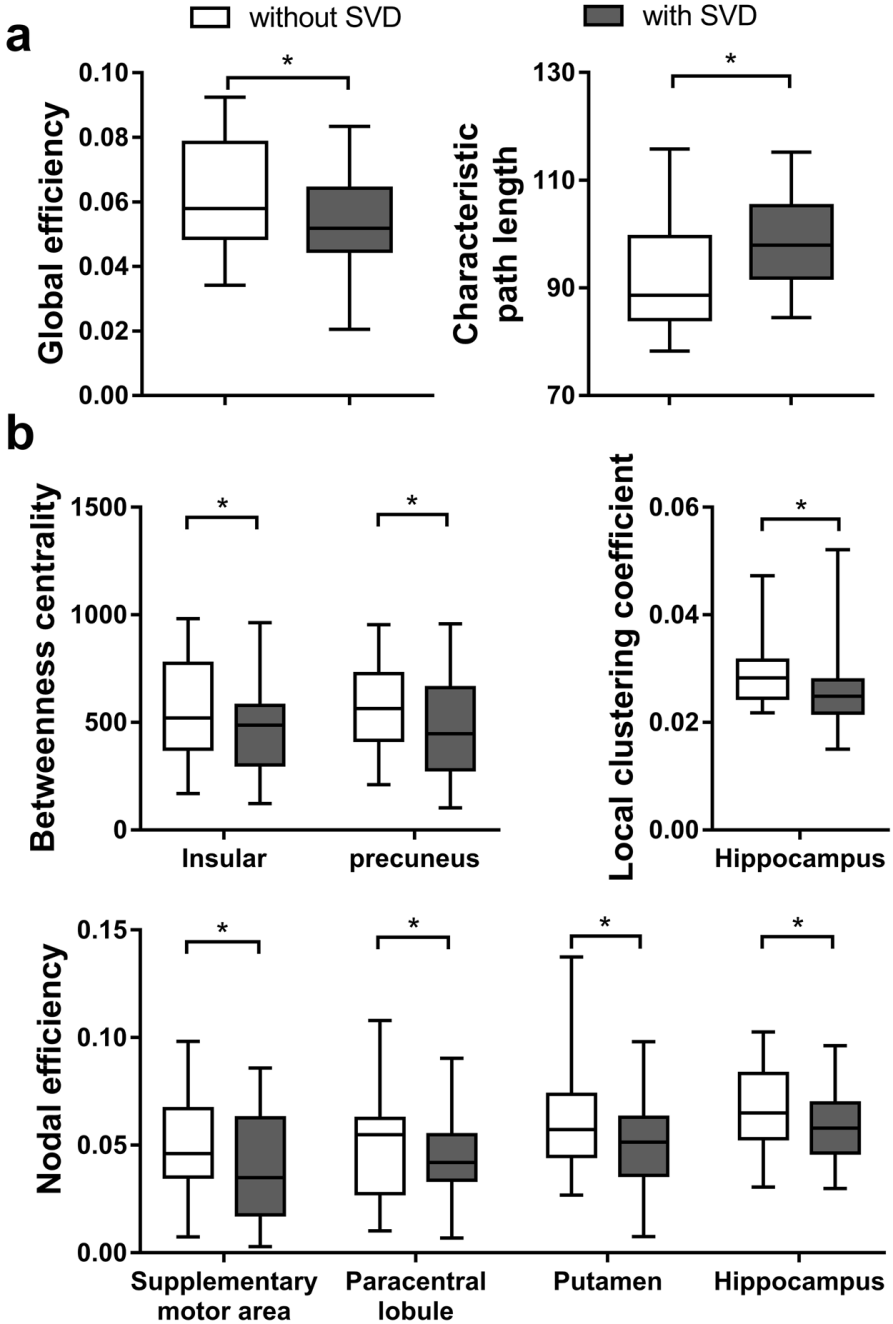
The measurement (mean + standard deviation) of the measures of the **(a)** global and **(b)** local structural brain networks of patients with first TIA or ischemic stroke. Note that only the measures that are significantly different between the two cohorts are shown. *p < 0.05.

The structural brain network of the two patient cohorts are shown in Fig. 1. They resemble the properties of small-world network with larger clustering coefficient (patients without SVD: 2.90 ± 0.43; with SVD: 3.03 ± 0.63) and equivalent characteristic shortest path length (without SVD: 1.25 ± 0.03; with SVD: 1.26 ± 0.04) as compared to random network. Figure 2 shows the measurement of network measures that are significantly different between the two cohorts. The characteristic path length (p = 0.003) and global efficiency (p = 0.005) of the brain network of patients with SVD are respectively higher and lower than patients without SVD (Fig. 2a).

The betweenness centrality of insular (p = 0.039) and precuneus (p = 0.036), and the local clustering coefficient of hippocampus (p = 0.005) of SVD patients are lower than patients without SVD. The nodal efficiency of supplementary motor area (p = 0.021), paracentral lobule (p = 0.045), putamen (p = 0.048) and hippocampus (p = 0.021) of patients with SVD are also lower. The two cohorts have the same set of brain hubs, namely, insular, precuneus, hippocampus, putamen, superior frontal gyrus, caudate, thalamus, globus pallidus, lingual gyrus.

### Association between total SVD score versus brain networks

**Figure 3:**
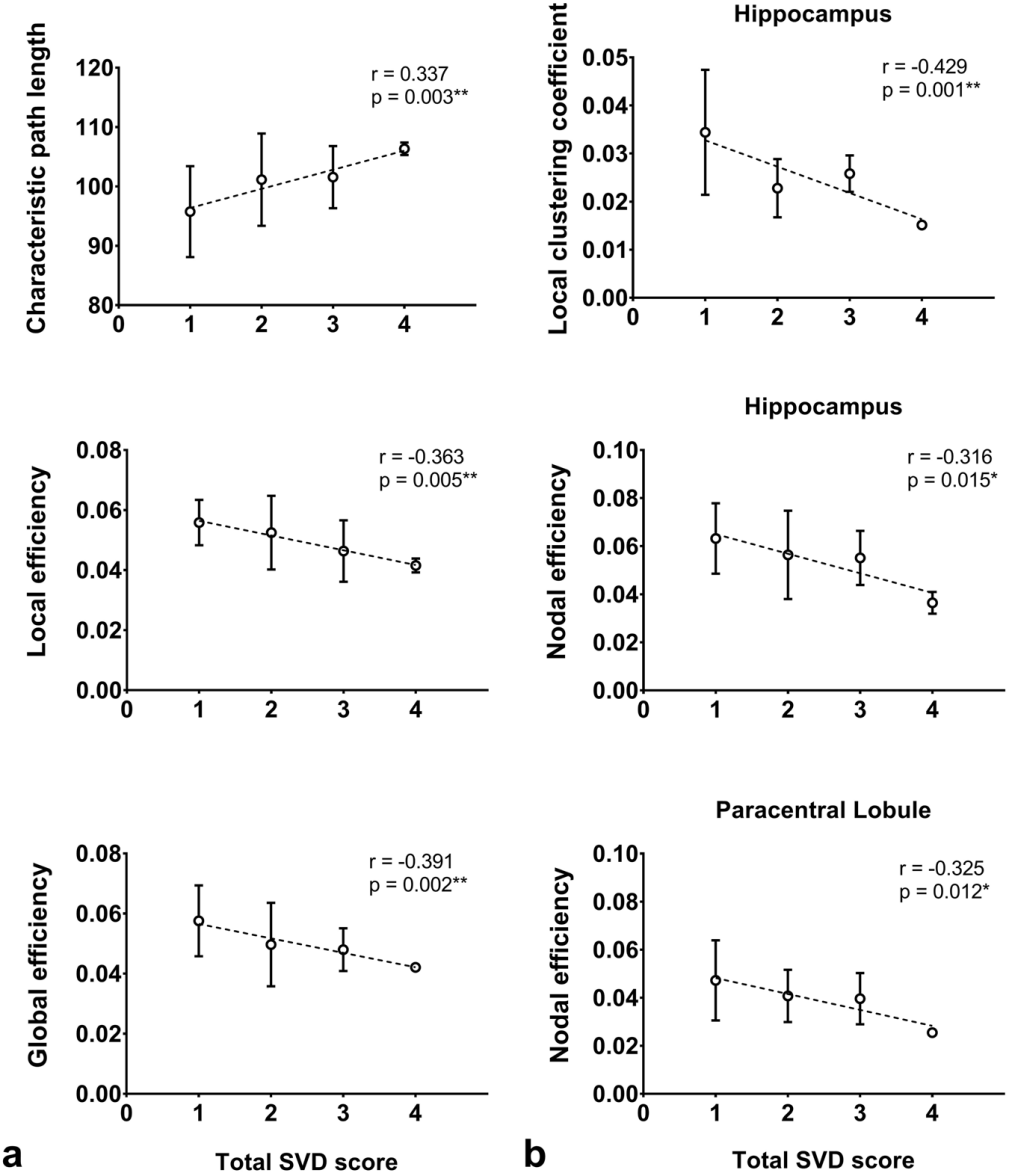
Relation between the measures (mean + standard deviation) of the **(a)** global and (**b**) local brain network versus the total SVD score for patients with SVD. Spearman rank correlation was performed to test association. The number of patients with total SVD score from 1 to 4 are 30, 19, 8 and 2, respectively.

The characteristic path length (r = 0.337, p = 0.003), global efficiency (r = −0.391, p = 0.002) and local efficiency (r = −0.363, p = 0.005) of the brain network of patients with SVD are associated with the total SVD score (Fig. 3a). The local clustering coefficient (r = −0.429, p = 0.001) and nodal efficiency of hippocampus (r = −0.316, p = 0.015), as well as the nodal efficiency of paracentral lobule (r = −0.325, p = 0.012) are also associated with the total SVD score (Fig. 3b).

## Discussion

With brain connectivity analysis, brain network can be systemically characterized at the global scale^20^. Normal brain has a high capacity for global information flow, also known as functional integration, for supporting cognition and behavior thanks to the underlying large-scale structural brain network that is characterized by high global efficiency and short characteristic path length^26^. We have demonstrated in this study that such optimal structural network architecture is disrupted in patients with SVD (Fig. 2). That the key consequences of confluent white matter lesions, such as periventricular and deep WMHI, are demyelination and axonal loss of the long projection fibers^27^ suggests that SVD likely has significant bearing on the long-distance structural connections between clusters of brain regions^28^. Global network changes similar to our study were also demonstrated by Lawrence *et al*.^29^ whom showed that the global structural brain network of patients with SVD was impaired, and the extent of which was related to cognitive impairment. In another study, patients with SVD who developed incident all-cause dementia in 5-year follow-up were found to exhibit lower global efficiency than SVD patients without dementia, and that the risk of incident dementia was independently and negatively associated with global efficiency^30^. The presence of multiple CMBs was also reported to alter the characteristic path length and global efficiency of patients with Alzheimer’s disease^25^. Other neurological diseases or disorders that reduce the global efficiency of the structural brain network include Alzheimer’s disease^31^, multiple sclerosis^32^ and schizophrenia^33^. Reduction of global efficiency was found to be associated with poorer behavior^32^ and cognitive^31^ performance.

Brain connectivity analysis also allows the characterization of the components of brain network at the local scale. The relation between a single brain region and its neighborhood can thus be investigated^34^. We have observed that various nodal characteristics, which reveal the communication capacity of a brain region with its neighborhood^35^, of hippocampus, precuneus, insular, supplementary motor area, paracentral lobule and putamen were reduced due to SVD (Figs 1 and 2). These brain regions are largely responsible for spatial and long-term memory^36^, higher-order cognitive functions^37^, and motor functions^38^. Structural disconnections in the local network of these brain regions may underscore the cognitive and motor impairments that are typically observed in patients with SVD^2^. The role of some of these brain regions has been implicated in previous studies^39,40^. For example, the volume of hippocampus is found to be related to the development of incident dementia^41^, and the cortical thickness of supplementary motor cortex to cadence^39^. In a different study of age-related cognitive decline, the nodal efficiency of insular is related to visuospatial function, and that of precuneus to executive function^42^.

The distribution of nodal degree, the number of connections that a node has, is ‘heavy-tailed’ for human brains^43^. In other words, there are only a small number of brain regions, known as hubs, that have large number of structural connections to other regions, and that they are essential to efficient global brain communication^43^. Disruption of the characteristics of which is previously shown to be associated with brain dysfunction^44^. That we observed no change in brain hubs due to SVD likely suggests that the overall impact of SVD on hub organization is limited.

Since different neuroimaging biomarkers of SVD often coexist, the total SVD score should provide a better assessment of the overall severity of SVD than an individual MRI biomarker^11^. Our findings (Fig. 3) showed that the overall SVD severity has insidious effect on most of the measures of the global network, and the local clustering coefficient and nodal efficiency of hippocampus, as well as the nodal efficiency of paracentral lobule. The total SVD score can explain up to 15% of the variance of the global network measure, and up to 18% and 11% of the variance of the local network measure of hippocampus and paracentral lobule, respectively. Our findings on the global network are similar to a previous study of cerebral amyloid angiopathy which showed an association between the overall SVD severity, measured using a different score, and global efficiency, with the former explaining only 7% of the variance of the latter^45^. In two other studies, relations between global network and individual SVD lesions, such as WMHI, lacunar infarcts and CMB, were demonstrated^29,46^. The global efficiency was also found to mediate the association between SVD lesions and cognition^46^.

In the main, our results (Figs 2 and 3) suggested that the capacity of a structural brain network for functional integration may be most vulnerable to change in SVD burden. So may the case for the communication of the entire brain network with hippocampus and paracentral lobule as well as the connections between the neighboring regions of hippocampus be. The role of SVD lesions on the neurodegeneration within hippocampus, a structure that is vulnerable to Alzheimer’s Disease, has been implicated^47^. Freeze *et al*. have demonstrated an interaction effect between cerebral amyloid-beta protein deposition, an Alzheimer’s Disease pathology, and SVD pathology on hippocampal neurodegeneration only when there is abnormal amyloid-beta deposition, suggesting that patients with the two pathologies are at higher risk of faster disease progression. Together with our results, it may be plausible that the disruption of the network connected to and the subnetworks around hippocampus due to SVD lesions are exacerbated by that due to amyloid-beta deposition.

A major limitation of our study is the lack of cognitive and behavioral assessments that could allow us to study the relation amongst the overall SVD severity, structural brain network and brain functions. Such relation was previously demonstrated^29,46^ but was limited to individual MRI biomarker of SVD, not total SVD score, for patients with large overall SVD burden. In addition, although deterministic tracking algorithm was the conventional method for estimating the edges of structural brain network^29,32,48^, it has several limitations, chief of which is its failure to resolve fiber crossing. The recently developed probabilistic tracking methods are more robust to fiber crossing, but may also yield spurious connections^49^. More advanced methods such as high angular resolution diffusion imaging and diffusion spectral imaging could be used to potentially resolve these issues in future studies. We also plan to investigate the relation between total SVD score and the change in the coupling between structural and functional brain networks in future studies in a hope to elucidate the underpinnings of the brain functional changes due to SVD.

## Conclusion

We have successfully demonstrated that SVD lesions disrupt both global and local structural brain networks for patient with TIA or first-time stroke. We also showed that the overall SVD severity has insidious effect on most of the measures of the global network, and the local clustering coefficient and nodal efficiency of hippocampus, as well as the nodal efficiency of paracentral lobule.

## Material and Methods

### Participants

We retrospectively reviewed the clinical and MRI data of consecutive patients admitted due to acute onset of neurological symptoms, and informed consent was obtained from all subjects. Patients with intracerebral hemorrhage, prior history of ischemic stroke, cerebral tumor, and other non-cerebrovascular neuropsychological conditions were excluded. A total of 95 patients with confirmed diagnosis of first TIA or acute ischemic stroke were included. Of these, 59 were found to have SVD lesions (see Total Small Vessel Disease Score below for how each type of SVD lesions was defined), and a corresponding control group of 36 patients without any SVD related lesions were included by matching age, gender and confirmed diagnosis of either TIA or first-time acute stroke. The classification of acute ischemic stroke was recorded based on the Trial of Org 10172 in Acute Stroke Treatment (TOAST)^50^. All procedures were carried out in accordance with operational guidelines of Human Research Ethics Committee, and all protocols were approved by the Institutional Review Board of the University of Hong Kong/Hospital Authority Hong Kong West Cluster.

### Image acquisition

MRI was performed using a 3.0 T MRI scanner (Achieva TX, Philips Healthcare, Best, The Netherlands) with body coil for excitation and 8-channel head coil for reception. For each subject, diffusion tensor imaging data, consisting of non-diffusion-weighted image (b0) and diffusion-weighted images (DWIs) with b-values = 1000 s/mm^2^ along 32 gradient directions, were acquired using single-shot echo-planar-imaging sequence with the following parameters: TR/TE = 4000/81 ms, field of view = 230 × 230 mm^2^, reconstructed resolution = 3 × 3 mm^2^, 33 contiguous slices with thickness of 3 mm, SENSE factor = 2, number of averaging = 2, total scan time *≈* 5 minutes. For anatomical reference, inversion recovery T1-weighted images were acquired with the following parameters: TR/TE/TI = 2000/20/800 ms, field of view = 230 × 197 mm^2^, reconstruction resolution = 0.75 × 0.8 mm^2^, 25 slices with thickness of 5 mm and gap of 0.5 mm, scan time = 2 min 50 s. Susceptibility-weighted images (SWI) were acquired using: TR/TE = 28/23 ms, field of view = 228 × 200 mm^2^, reconstruction resolution = 0.89 × 0.78 mm^2^, 135 contiguous slices with thickness of 1 mm, scan time = 3 min 32 s. Axial T2-weighted images with the same geometry as that of diffusion MRI acquisition were obtained using multishot-turbo-spin-echo sequence with the following parameters: TR/TE = 3000/80 ms, field of view = 230 × 180 mm^2^, reconstruction resolution = 0.33 × 0.33 mm^2^, 25 slices of with thickness of 3 mm and gap of 0.5 mm, scan time = 1 min 18 s. Fluid-attenuated inversion recovery (FLAIR) images were acquired with following parameters: TR/TE = 4800/263 ms, field of view = 230 × 202 mm^2^, reconstruction resolution = 1.2 × 1.2 mm^2^, 60 contiguous slices with thickness of 2.5 mm, scan time = 4 min 33 s.

### Total small vessel disease score and infarct quantification

Both CMBs and lacunar infarcts were assessed based on the international consensus^4^. Focal lesions that were rounded, well-defined and hypointense with diameter less than 10 mm on SWI were considered as CMBs. CMBs mimics, such as blood vessel, mineralization in the globi pallidi or dentate nuclei, hemorrhage within infarct or small hemorrhage close to large infarct, air-bone interface and partial volume artifact, were excluded. Lesions that were round or ovoid with diameter between 3 and 20 mm, and signal intensity similar to CSF on FLAIR images were considered as lacunar infarcts^51^. WMHI were assessed based on the Fazekas scale^52^. Periventricular WMHI with Fazekas score of 3 (irregular periventricular signal extending into the deep white matter) and/or WMHI in deep brain with Fazekas score of 2 (confluent areas) were considered as high-grade WMHI^4^. PVS were defined as punctate or linear hyperintense lesions smaller than 3 mm in diameter on T2-weighted images, and were rated on a semi-quantitative scale from 0 to 4^10^. Moderate to severe (scale 2–4) PVS in the basal ganglia was defined as high-grade PVS^4^. The total SVD score was subsequently obtained by giving one point each to the presence of lacunar infarct, CMBs, high-grade WMHI, or high-grade PVS^12^. Patient with total SVD score higher than 0 was assigned to the SVD cohort.

Multi-slice binary mask of the ischemic infarct for each patient was manually defined on the mean of all DWIs with b-values = 1000 s/mm^2^. Only pixels with intensity distinctly higher than those in the contralesional hemisphere were included in the infarct mask.

### Image processing and brain network construction

The image pre-processing steps for brain connectivity analysis were performed using the FMRIB’s Software Library (FSL) version 5.0^53^.

#### Brain parcellation

T1-weighted images were used for parcellating the brain into 90 cortical and subcortical regions based on the Automated anatomical labeling (AAL) atlas with the following steps: (1) T1-weighted images were first coregistered to DWIs in the native space using FLIRT of FSL^54^. (2) The coregistered T1-weighted images were subsequently mapped to the ICBM 152 template using FNIRT of FSL^54^. (3) Estimated transformation parameters were inverted and applied to the AAL atlas to warp all brain regions-of-interests from the MNI space to the native diffusion space.

#### White matter tractography

Corrections for eddy current and head motions were performed by coregistering all DWIs to b_0_ using FMRIB’s Diffusion Toolbox^53^. Diffusion Toolkit^55^ was also used to estimate the diffusion tensor using the linear least-squares fitting method. To construct the structural connections between the 90 brain regions, deterministic whole-brain fiber tracking was performed using the Fiber Assignment by Continuous Tracking (FACT) algorithm of TrackVis (http://trackvis.org) with fractional anisotropy threshold of 0.2 and turning angle threshold of 45°.

#### Brain network

In a brain network, each brain region is considered as node whilst the connections between regions as edge. Structural connections between two regions are considered as edges only when WM fiber tracts originate from one region and terminate in the other. The number of fibers between regions was then used to weight each edge using the UCLA Multimodal Connectivity Package in the native diffusion space^56^. An inter-regional undirected network with weighted connections was subsequently obtained for each subject.

### Brain connectivity analysis

Each weighted connectivity matrix was normalized to its maximum fiber count to minimize the overall differences in the connectivity strength between patients^20^. The characteristics of global network, such as the properties of small-world network and network efficiencies, as well as those of the local network for each brain region, also known as nodal characteristics, for each subject were estimated using the Brain Connectivity Toolbox^20^. For a detailed review on the measures of brain network, please refer to ref.^20^.

#### Small-world properties

Measures of small-world network were first introduced by Watts and Strogatz^57^. Clustering coefficient of a node, also known as local clustering coefficient, measures the likelihood that its neighborhoods are connected to each other. The clustering coefficient of a network reflects the extent of local cluster in that network. Thus, network with high clustering coefficient has high level of functional segregation. Characteristic shortest path length measures the shortest geodesic length between a node and any other node. The characteristic shortest path length of a network is the average of the shortest path length of all pairs of nodes in that network. Network with low characteristic shortest path length promotes parallel information propagation for functional integration. To determine whether a network is a small-world network, the clustering coefficient and characteristic shortest path length of the network are compared to 100 random networks that are generated based on the same number of nodes, edges and degree distribution as that of the network-of-interest^20^. A small-world network has normalized clustering coefficient larger than one and the normalized characteristic shortest path length close to one^57^.

#### Network efficiencies

Global efficiency is computed as the average of the inverse of the shortest path length of all node pairs in a network^58^. It estimates the ability of a network for integrating and coordinating information. Local efficiency of a network can be estimated by averaging the global efficiency of all subnetworks. It measures how well each subnetwork exchanges information when the most-connected node in the subnetwork is removed^59^.

#### Nodal characteristics

The degree of a node is the number of edges connecting it to rest of the network^20^. Nodal efficiency of a node is defined as the inverse of the harmonic mean of the characteristic shortest path length between other nodes in a network and itself^34^. It reflects the importance of the node for communication within a network. The betweenness centrality of a node quantifies the number of shortest paths between other nodes in an entire network and itself. It is a measure of the importance of the node for the integration of all the connections in a network^35^.

#### Identification of hubs

Brain regions that play key roles in the coordination of information flow in a network are considered as hubs. Brain hubs are characterized by their large number of connections or high degree of connectivity to other regions^43^. They occupy a central placement in the network and contribute significantly to functional integration. To identify hubs, brain regions are ranked by hemisphere-averaged betweenness centrality and degree. The two ranks are then summed to obtain a hub score. Brain regions with the top 20% score are considered as network hubs^60^.

### Statistical analysis

Sex, history of diabetes mellitus, hypertension, ischemic heart disease and smoking, presence of infarct in 7 brain areas, namely basal ganglia, brain stem, cerebellum, frontal lobe, parietal lobe, temporal lobe and occipital lobe, and treatment of antiplatelets and lipid-lowering drugs were recorded as binary variables. Stroke classifications were recorded as categorical variable. Age, infarct size and serum total cholesterol were recorded as continuous variables. Demographics were compared between patients with and without SVD using independent samples t-test for continuous variables and Fisher’s exact test for proportions. The nodal characteristics for each brain region from the two hemispheres were averaged before statistical analyses. Independent-samples t-test were performed to evaluate the group difference in all global and local network measures between patients with and without SVD. The association between all network measures and total SVD scores of patients with SVD were performed using Spearman rank correlation. All analyses were adjusted for age, sex, stroke classification, infarct size and location, and vascular risk factors (hypertension, diabetes, serum total cholesterol). Bonferroni correction for the problem of multiple comparisons was performed for all statistical analyses described above. SPSS 22.0 (SPSS, Chicago, IL) was used for all the statistical analyses. A significance level of p < 0.05 was set for all statistical tests.
